# Dora's mother: a housewife's psychosis

**DOI:** 10.1192/bjb.2022.64

**Published:** 2023-08

**Authors:** Anuradha Menon

**Affiliations:** Becklin Centre, St James's University Hospital, Leeds, UK

**Keywords:** Hysteria, Dora, Freud, syphilis, mother

## Abstract

I examine a speculative diagnosis made by Sigmund Freud regarding his patient's mother in his landmark 1905 paper describing a hysterical illness. Freud considered the impact of Dora's mother's mental state on her daughter, wondering whether the mother might suffer from a ‘housewife's psychosis’. Here was an emphasis on the social structures of the times and differences between the parents in terms of sexual freedom and societal limitations placed on women. Freud's description drew attention to Dora's anxieties in relation to her parents, in particular the state of their sexual relationship and the apparently sanctioned entry of another couple, Frau and Herr K, into the parental relationship. In particular, the role of syphilis in the aetiology of sexual disturbances was considered, affecting men and their sexual partners, specifically their wives, who faced lifelong risks of morbidity, inadequate treatment and psychic disturbances at this time in 19th century Vienna.

The case of Dora,^1^ through which Freud outlined his theory of hysteria and came to understand the power of transference, pre-dated many important discoveries in psychoanalysis: the role of transference and countertransference in a treatment, the structural theory, the dual instinct theory, the second theory of anxiety, as well as a host of other significant later advances.^2^ It is for us then, reading the case more than 100 years on, to admire Freud's ingenuity in trying to enter imaginatively into a young woman's complicated motives for ‘choosing’ a neurosis. In doing so, he shows us a way of entering the world of the intrapsychic, which, as we know, deepens clinical understanding and provides meaning to a socio-politico-cultural^3^ formulation.

On reading the Dora case as a training psychiatrist, I was always fascinated by a seemingly minor detail: a throwaway diagnosis of Freud's regarding Dora's *mother's* strange illness. The speculative diagnosis regarding this ‘unseen’ member of the patient's family was made by Freud, remotely, from reports made by others. Freud's brief observations, sensitive and emphatic by turns, began to sketch out the sort of mother that Dora had:

‘an uncultivated woman and above all a foolish one […] who had concentrated all her interests upon domestic affairs, especially since her husband's illness and the estrangement to which it led.’^1^

Before I examine his point, I briefly summarise below an outline of the Dora case.

## The ‘Dora’ case

Dora was analysed by Freud for 3 months in the autumn of 1900. Aged 18, she was brought by her father to Freud with a series of mental and physical symptoms – depression and difficulty socialising, along with a nervous cough, a loss of voice, migraines and difficulty breathing. Significantly, her father had himself been treated some years ago by Freud for syphilis, and then for some years he had suffered various other illnesses. Dora's previously idealised view of her father had turned hostile, and her relationship with her mother was full of conflict. While sifting through reports of emotional upsets and suicidal behaviour, Freud noticed the family's concurrent relationship with a young couple, Herr and Frau K, pre-dating the onset of Dora's symptoms. Here Freud began to develop his theory of the formation of Dora's hysterical symptoms. The first was a ‘reversal of affect’, where sexual excitement was turned into disgust, and the second ‘displacement of sensation’, where a stimulus felt in one part of the body, and felt to be unacceptable, was repressed and relegated to another, unrelated area. Freud described the hysterical symptom as resulting from a path taken by the psychic conflict into the body: a somatic compliance. Two dreams that emerged in treatment were analysed following what was by now an established dream interpretation technique.^4^ They revealed a conflict and repressed desires: Dora's adolescent preoccupation with and terror of sexual intimacy, resulting in repression of her sexual feelings, as well as her unconscious desire for revenge against her beloved father, who was felt to have betrayed her. The idea of the hysterical symptom being the outcome of an irresolvable sexual conflict was thus clearly shown through a case-history format. Dora's fantasies of being of central importance in the family drama, followed by bitter disappointments, made hysteria her ‘solution’ to the internal dilemma. Freud wrote ‘incapacity for meeting a real erotic demand is one of the most essential features of a neurosis. Neurotics are dominated by the opposition between reality and phantasy’.^1^

Among Freud's five case histories, Dora's stands out as probably the most discussed and critiqued by his successors.^5^ Blass writes ‘Through Dora's analysis Freud gradually passes from the seduction theory to a theory of neurosis that centres on intrapsychically originating moral conflict and on the early childhood events that make it pathogenic’.^6^

There were, however, several clinical discoveries which Freud considered later, looking back on the work: Dora's underlying homoerotic conflicts, her transference to Freud, the countertransference, the brevity of the treatment. Freud's Oedipal theory was yet to be fully developed.^6^ Conversion came to be understood as the body becoming the stage on which an unacceptable conflict in the psyche gets staged or represented, and this mechanism was viewed as radically different from that of hypochondria and severe psychosomatic illnesses. A detailed exposition of these ideas in contemporary psychoanalysis is beyond the scope of this paper but interested readers are encouraged to read further.^7^

## Dora's mother: the background

Freud suggested that ‘a daughter takes her mother's love story as her model’. Dora's mother had met her future husband, a man much older than her and sexually experienced, at 17 and then waited 2 years to marry him. As a mature woman and mother, she was now exacting, preoccupied with looking after material household things and seemingly indifferent to Dora's emotional needs. She also seemed strangely cut off from evidence of her husband's infidelity, offering disinterested explanations in response to the anxious questioning of her precocious daughter. Dora was anxious about her father's relationship with Frau K, a younger, married woman and a family friend:

‘ … whenever she had reproached her father about Frau K., he had been in the habit of saying that he could not understand her hostility and that, on the contrary, his children had every reason for being grateful to Frau K. Her mother, whom she had asked for an explanation of this mysterious remark, had told her that her father had been so unhappy at that time that he had made up his mind to go into the wood and kill himself, and that Frau K., suspecting as much, had gone after him and had persuaded him by her entreaties to preserve his life for the sake of his family. Of course, Dora went on, she herself did not believe this story; no doubt the two of them had been seen together in the wood, and her father had thereupon invented this fairy tale of his suicide so as to account for their rendezvous.’^1^

There was clearly an unhappy, heavily disguised family drama in the backdrop of Dora's hysterical presentation. Her suicidally depressed father and rather cut-off mother seemed to have relied on Frau K to act as an intermediary in their relationship. Freud correctly gauged Dora's scorn at her mother's naivety in taking the father's account of his relations with Frau K at face value. Dora presented with hysterical symptoms alongside her conscious contempt of both her parents for their tendency to self-deception and lies.

Freud noted at the start how Dora's father told him ‘you know I get nothing out of my own wife’ as a justification for his involvement with Frau K: a loaded sentiment ironically echoed by Herr K in describing *his* wife when he made sexual advances towards Dora. This acted as a trigger for Dora's hysterical symptoms. The subtext seemed that this was a world where men were justified in seeking their sexual pleasures freely and at some risk, as long as they kept up appearances to save face while married. Freud deftly linked these confusing observations in young Dora's mind, giving them a voice: ‘How dare the man make an assumption about me?’. A passing reference to her mother being in Herr K's confidence, hearing bitter complaints about Frau K's relations with Dora's father, made one wonder about her complicity in Dora's predicament.

What might be the mysterious illness that plagued Dora's mother all this while? Freud wrote:

‘She presented the picture, in fact, of what might be called the “housewife's psychosis”. She had no understanding of her children's more active interests and was occupied all day long in cleaning the house with its furniture and utensils and in keeping them clean – to such an extent as to make it almost impossible to use or enjoy them.’^1^

He noted that if the conditions were not suitable for a hysterical symptom, as was possibly the case with Dora's mother, one found instead a psychical symptom. She was caught up in a mindless trap of domesticity, posing a severe problem to her family:

‘This condition, traces of which are to be found often enough in normal housewives, inevitably reminds one of forms of obsessional washing and other kinds of obsessional cleanliness. But such women (and this applied to the patient's mother) are entirely without insight into their illness, so that one essential characteristic of an “obsessional neurosis” is lacking.’^1^

The lack of insight or understanding of the other and the repetitive acts which made it impossible for others to enjoy their comforts: all these seemed to be significant. Here I thought Freud was marking a lack of receptivity; instead, there seemed a marked tendency to repulse others, to keep them away from her person while keeping up a façade of housewifely devotion. This could be a projection into the other of a desperate need to get in, while thwarting this and erecting a psychic barrier. For example, Dora's father stated that he got ‘nothing out of’ his wife as a hint of a lack of sexual relations; this may be rephrased as ‘I cannot get into her’. This was also Dora's experience of an unavailable mother. Instead, her mother transformed the house, and her body, into a whirlwind of activity that did not allow for entry or habitation.

Freud then considered the role of heredity in hysteria, and gave Dora's mother a place in this constellation:

‘I do not wish to give an impression of underestimating the importance of heredity in the aetiology of hysteria or of asserting that it can be dispensed with. In the case of the present patient the information I have given about her father and his brother and sister indicates a sufficiently heavy taint; and, indeed, if the view is taken that pathological conditions such as her mother's must also imply a hereditary predisposition, the patient's heredity may be regarded as a convergent one.’^1^

## The role of neurosyphilis

One might speculate on another possibility that lent credence to Freud's observations of Dora's neurosis: her mother's ‘psychosis’ and the peculiar circumstances of a sexual (parental) couple of that time. Dora's mother had already contracted syphilis from her partner early in the marriage, like many middle-class women who seemed to have little choice other than to suffer the illness and seek treatment, risking shame and humiliation. Freud, in describing the significance of the male parents’ syphilis in their children's illnesses, is mysteriously silent on the mother's predicament:

‘To my mind, however, there is another factor which is of more significance in the girl's hereditary or, properly speaking, constitutional predisposition. I have mentioned that her father had contracted syphilis before his marriage. Now a strikingly high percentage of the patients whom I have treated psycho-analytically come of fathers who have suffered from tabes or general paralysis. In consequence of the novelty of my therapeutic method, I see only the severest cases, which have already been under treatment for years without any success. In accordance with the Erb-Fournier theory, tabes or general paralysis in the male parent may be regarded as evidence of an earlier luetic infection; and indeed I was able to obtain direct confirmation of such an infection in a number of cases. […] [T]he conclusion to which I have been driven by my experience as a neuro-pathologist [is that] that syphilis in the male parent is a very relevant factor in the aetiology of the neuropathic constitution of children.’^1^

The age of Freud was also the age of syphilis, as pointed out by Ropper & Burrell,^8,9^ while psychoanalysis grew into a body of work fuelled by Freud's emphasis on patients’ fantasies around sex. In a patient's psychic elaboration of phantasy, the patient's real experience was crucial as the shameful and terrifying spectre of syphilis hung over every sexual encounter like ‘the sword of Damocles’. By the late-19th century, neurologists acknowledged that syphilis was, in its pathological manifestations, the ‘great imitator’ of maladies.

Freud commented on Dora's father's luetic condition before marriage, as well as his successful involvement in treating the latter's troubling vascular and neurological syphilitic symptoms 4 years before Dora arrived as Freud's patient. But in fact, prior to the first use of penicillin against syphilis in 1943, it is well-known that mercury compounds had a prominent position in the medical practice despite a tremendous toxicity and a questionable efficiency.

Freud, with his background in neurology before his pioneering work in psychoanalysis, would have keenly observed syphilis's twofold impact: the neurological manifestation of syphilis, a ‘general paresis of the insane’, which galvanised biological psychiatry while the psychological impact gave impetus to the study of hysteria.^8^ Ironically, it was in Germany in 1905 that the discovery of the spiral-shaped bacterium now known as *Treponema pallidum* was made by zoologist Fritz Schaudinn and dermatologist Erich Hoffmann; this changed the understanding of syphilis and general paresis of the insane.

But the aspect of the family drama often got minimised: the terrible social and psychological impact of syphilis on the sexual relations of couples, an experience of unconscious threat, neglect and violence. Most significantly, a legitimate place for the mother's state of mind and for her sense of an interiority was lacking.

Freud speculated on a possible early transmission of Dora's father's syphilitic infection to his wife, as well as the possibility that he was later cured of his condition. But what of Dora's mother, who continued to cohabit with a man who may or may not have been cured of syphilis, may have been suffering tertiary syphilitic complications and whose further infidelity was betrayed by gonorrhoea? We do not know; Freud was never able to meet her, nor discuss what influence her situation might have had on Dora. We also do not know when the mother's venereal symptoms began or whether they were fully treated and cured; however, we can speculate on an experience of sex being fraught with danger. A phantasy of sex as destructive may have turned into reality, a fact that was perhaps not lost on Dora.^10^

Syphilis was widespread enough to give rise to a special form of hypochondria known as syphilis imaginaria, or the development of imaginary symptoms of syphilis.^11^ Physicians also reported syphilophobia, or the exaggerated fear of the disease, and hydrargyrophobia, or the fear of mercury, which was one of the main medical treatments.^12^

It is also difficult to know whether a ‘vertical’ transmission of syphilis from mothers to babies as congenital syphilis, causing still-births and miscarriages, was a possibility in Dora's mother's case before the birth of Dora and her brothers.

By the time Dora was brought by her father to see Freud, the parental couple had ostensibly ceased sexual relations – perhaps even as early as when she was 6 years old and her father contracted tuberculosis. Dora suggested to Freud at one point that her father was impotent. She hinted also of her knowledge of sexual activities involving other parts of the body if genital sex was inadvisable. Freud's elaboration of Dora's sexual fantasies, linking them to her hysterical symptoms, is at once a phenomenological enquiry into the individual psyche and a description of the social and family structures in 19th-century Vienna. And so he noted Dora's complaints against her father as a ‘wearisome monotony’ with an ‘incessant repetition’, at the knowledge of her father's extramarital affair. The sexual betrayal of the mother by the father thus seemed to have been experienced as something unbearable by Dora, and led to her behaving like a jealous wife, while her mother stayed silent.

If the ‘housewife's psychosis’ was a disturbed relationship with reality, Dora's mother's behaviour signalled an internal disturbance that could not be articulated. Taking up the syphilitic vector as a projected experience perhaps of ‘care-lessness’ originating in the father's apparent indifference allowed another way of looking at Dora's mother's frantic avoidance of contact. Freud had already suggested the possibility that shame and humiliation, a sense of being contaminated, gave rise to a reaction formation that drove Dora's mother's incessant cleaning. The sense of being ‘dirty’ seemed to be linked to the mother's venereal infection, the white vaginal discharge and abdominal pains which needed medical treatment. But the sense of neglect and damage felt to be inflicted by the other in this way was perhaps projected into Dora by her parents. In later emphasising the significance of Frau K in Dora's fantasies, Dora's conscious alienation from her mother and alliance with her father seemed to mask, at a deeper level, an unconscious longing for an unavailable mother and rivalry with a father who pursued his own needs at a heavy cost to the family:

‘The relations between the girl and her mother had been unfriendly for years. The daughter looked down on her mother and used to criticize her mercilessly, and she had withdrawn completely from her influence.’^1^

If Dora was perhaps forced to stand on the sidelines while her mother lavished attention on material things, she eventually found herself in a similar position while her father found substitutes for her mother. Freud's final observations of Dora's father as ‘never entirely straightforward’ and Dora's dropping out of analysis seemed to confirm this and has since been used (sometimes unfairly) as a way of underlining Freud's own contentious, academic motives in this unsatisfactorily brief yet trail-blazing treatment.

## Conclusions

Freud's academic interest lay in working out his theories of hysteria, but one wonders about his being taken aback by the mysterious ways in which female sexuality showed itself or remained obscure, finding its peculiar diversions in a patriarchal social order. The sociocultural aspect of syphilitic infection in the 1900s has been examined extensively,^8^ linking to femininity^13^ and the family matrix.^12^ Feminist writers argue that early psychoanalysts failed to fully acknowledge that, for many, the avoidance of sex with men was based on their fear of contracting venereal disease and, for married women, their fear of passing the disease to their children. Sexual relations outside the social contract of marriage carried dangers as well, with the added elements of guilt and punishment for transgression complicating treatment options for sexually transmitted illnesses. The secrecy and fear surrounding venereal disease influenced many social attitudes, reinforced the division between races and social classes, and significantly influenced the construction of female sexuality and the notion of family.^13^ Today we also speak of ‘reckless transmission’ of infection as a legal definition.^14^

However, this paper is more concerned with the *intrapsychic* as an inquiry into the internal forces driving the victim of a sexually transmitted infection when they contract the illness without their conscious knowledge. It is possible such a victim may feel paralysed by the threat of being invaded and taken over from the inside by something that ‘catches’, and if so, the notion of sex being dichotomised as either safe or transgressive, by both the individual and society, comes into question. One such example is a research project in the UK into women with a range of sexualities, living with HIV/AIDS.^15^ The findings highlight the woman's sense of being unheard, the stigma and the barriers, both internal and external, faced when she contracts a sexually transmitted infection from an infected partner and requires help and treatment. The report, drawing on focus groups and online surveys, highlights how women are often described as ‘hard to reach’ and yet there is a lack of clear guidelines regarding emotional support for sufferers. The spread of HIV among women has sociopolitical as well as biological roots: it arises from the inequity between the sexes, societies’ class structures and the inaccessibility of healthcare. In many societies, educational and employment opportunities for women are limited. Throughout the world, women are placed in subservient positions and lack the freedom to ask questions or to demand the use of condoms.^16^ Perhaps here, like with syphilis in the 19th century, there is still a considerable gap in understanding a patient population defined by its position in the background of the ‘actual’, or identified, disease victims.

Dora's mother, like Shakespeare's sister in academia,^17^ may be always at risk of being relegated to obscurity in a patriarchal social setting. This is perhaps unavoidable, though one missing link may be the spectre of syphilis and its psychological impact on women of that time, with its inevitable impact on the sexual couple. But in the wonderful rapidity and prescience of his work, Freud himself does give shape to Dora's mother as a shadowy yet significant player in Dora's 19th-century teenage world, assigning a diagnosis of a ‘housewife's psychosis’ – a retreat from reality into mad domesticity as a defence, carrying with it a wealth of possible meanings for retrospective investigation.

## Data Availability

Data availability is not applicable to this article as no new data were created or analysed in this study.

## About the author

**Anuradha Menon** is a psychoanalyst and consultant in liaison psychiatry and medical psychotherapy in the Becklin Centre at St James’s University Hospital, Leeds, UK.
